# Análisis de los valores de hipertensión arterial en pacientes que emplean sistemas personalizados de dosificación

**DOI:** 10.1016/j.aprim.2021.102251

**Published:** 2021-11-26

**Authors:** Adela Martín Oliveros, Coral García-Pastor, Jesús Carlos Gómez Martínez, Noelia Tejedor-García

**Affiliations:** a Farmacéutica comunitario Córdoba, miembro de la Sociedad Española de Farmacia Clínica Familiar y Comunitaria (SEFAC), Córdoba, España; b Farmacéutica comunitario Madrid, miembro de la Sociedad Española de Farmacia Clínica Familiar y Comunitaria (SEFAC), Madrid, España; c Farmacéutico comunitario Barcelona, miembro de la Sociedad Española de Farmacia Clínica Familiar y Comunitaria (SEFAC), Barcelona, España; d Universidad Francisco de Vitoria, miembro de la Sociedad Española de Farmacia Clínica Familiar y Comunitaria (SEFAC), España

Existe un grave problema de adherencia en ancianos hipertensos, lo que contribuye a que solo el 30% consiga controlar sus valores de hipertensión arterial (HTA)^1^. Los sistemas personalizados de dosificación (SPD) podrían mejorar la adherencia^2^, ^3^ así como el control de la hipertensión, por lo que se comparó la evolución de presión arterial sistólica/presión arterial diastólica (PAS/PAD) en usuarios no adherentes de SPD frente a un grupo control que no los empleaba.

Con el objetivo de analizar la adherencia y los niveles de presión arterial en pacientes no adherentes con HTA no controlada, polimedicados y mayores de 55 años tras el empleo de SPD, se diseñó un estudio epidemiológico, multicéntrico en el que participaron 35 farmacias, 17 en el grupo control y 18 en el grupo SPD. Se reclutaron 107 y 88 pacientes, respectivamente. Se realizó una visita inicial, al mes, tres meses y seis meses. Todos los pacientes permanecieron hasta el final del estudio y no había diferencias significativas entre ambos grupos al comenzar el mismo. Se analizaron datos sociodemográficos, comorbilidades, antecedentes, ingresos hospitalarios, fármacos antihipertensivos y otros fármacos prescritos. Los criterios de inclusión fueron ser no adherente a la medicación, HTA no controlada, polimedicación, ser mayor de 55 años y emplear la receta electrónica desde al menos tres meses antes de incluirse en el estudio.

Se analizaron los niveles de adherencia al tratamiento mediante test de Morisky-Green, contaje de medicación devuelta (en el grupo SPD) y valores de presión arterial (PA). La PA se midió con un tensiómetro validado por la *European Society of Hypertension* (ESH) siguiendo el protocolo establecido en las guías internacionales^4^.

La adherencia aumentó en ambos grupos desde la primera visita (100, 100 y 98,9% en el grupo SPD vs. 92,5, 96,3 y 95,3% en el grupo control al mes, tres meses y seis meses respectivamente). Sólo se encontraron diferencias significativas entre ambos grupos en la visita realiza al mes (valor p entre grupos = 0,00087)

El número de principios activos prescritos inicialmente para el tratamiento de la HTA era de 4,6 (± 3,6) en el grupo SPD y 3,5 (± 2,2) en el grupo control (p = 0,007). En el grupo SPD hubo un descenso significativo (p < 0,0001) desde el primer mes que se mantuvo hasta los seis meses (2,7 ± 1,4, mes 1; 2,6 ± 1,3, mes 3y 2,6 ± 1,4, mes 6). No hubo diferencias significativas con el grupo control (2,6 ± 1,3, mes 1; 2,6 ± 1,3, mes 3 y 2,7 ± 1,3, mes 6).

La PAS mostró un descenso, entre la primera visita y los seis meses, de 18,3 (± 14,3) mmHg (p < 0,001) en el grupo SPD y 9,9 (± 11,0) mmHg (p < 0,001) en el grupo control. A los seis meses se encontraron diferencias significativas entre ambos grupos (p = 0.002) (fig. 1 superior). La PAD descendió, entre la primera visita y los seis meses, 11,49 (± 15,6) mmHg en el grupo SPD (p < 0,001) y 8,9 (± 11,8) mmHg en el grupo control (p < 0,001); no hubo diferencias significativas entre ambos grupos (p = 0,540) (fig. 1 inferior).

**Figura 1:**
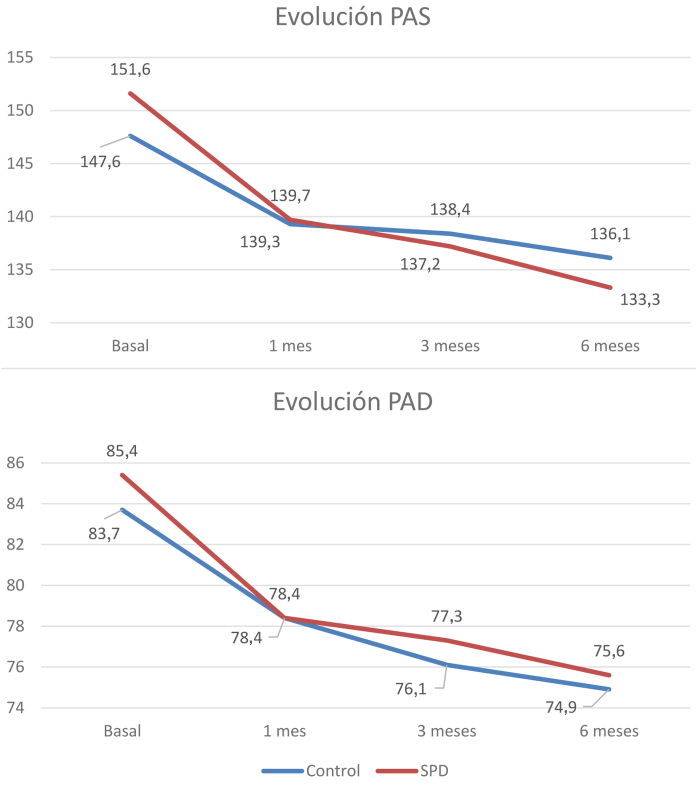
Evolución de la presión arterial sistólica y diastólica según grupo de estudio.

Un 47,7% de los pacientes del grupo SPD alcanzaron el control de la presión arterial a los seis meses vs. un 39,3% del grupo control. Este aumento fue únicamente significativo en el grupo SPD (comparando la visita al mes y a los seis meses).

El grupo SDP consiguió una mayor reducción de la PAS con un menor coste de medicación antihipertensiva (109,34 € vs. 201,03 €). Este resultado no incluye el coste de tiempo del farmacéutico para preparar el SPD que se estimó en 114,98 € (IC 95%: 99,32€ a 130,65€) y el coste de las ampollas (12,9 € por seis meses).

Como conclusión se puede deducir que el empleo de SPD supuso una mejora de los niveles de PA de los pacientes por lo que su uso se postula como una buena herramienta (costo-efectiva, bien tolerada por los usuarios y de fácil uso) para mejorar la adherencia de los pacientes y controlar su HTA, aunque son necesarios más estudios.

## Responsabilidades éticas

El trabajo fue aprobado por el Comité de Ética de Investigación regional de la Comunidad de Madrid. Los autores recogieron el consentimiento informado de todos los participantes.

## Financiación

Este estudio ha sido financiado por los laboratorios Mylan.

## Conflicto de intereses

Los autores declaran no tener ningún conflicto de interés
